# Facial Nerve Palsy after Inferior Alveolar Nerve Block: A Rare Presentation of Ocular Complication and Literature Review

**DOI:** 10.3390/reports6040047

**Published:** 2023-10-04

**Authors:** Glauco Chisci, Dafne Chisci, Enea Chisci, Viola Chisci, Elettra Chisci

**Affiliations:** 1 Centro Dentistico Chisci, Via Ricasoli 18, 58100 Grosseto, Italy; 2 Department of Dentistry, University of Milano-Bicocca, 20126 Monza, Italy; 3 Department of Ophthalmology, University of Palermo, 90121 Palermo, Italy; 4 Department of Ophthalmology, Greifswald University Hospital, 17489 Greifswald, Germany

**Keywords:** anesthesia, bell palsy, dental anesthesia, dental injection, endodontics, facial nerve palsy, facial palsy, inferior alveolar block, ocular complication, ophthalmic complication, optocaine

## Abstract

Many ocular complications are described in the literature after dental injections. Facial nerve palsy is a rare complication. We report a case of a 60-year-old woman in the American Society of Anesthesiologists (ASA) I classification under orthodontic treatment with aligners that required an inferior alveolar block for endodontic treatment. Optocaine with epinephrine 1:200,000 and a disposable needle 25 G × 36 mm mounted on a dental syringe were used, and the effect of the anesthesia arose after 10 min. Facial nerve palsy on the side of the injection arose after 1 h and 40 min from the injection, and the patient was immediately visited by an ophthalmologist who reported the examination reported in the present case report. The authors report this unusual case with a subsequent onset and short duration of facial nerve palsy and discuss possible anesthetic solution pathways.

## 1. Introduction

A local anesthetic injection is a common procedure performed in dentistry. Despite the evaluation of the patient’s medical history, anesthetic selection and proper tissue preparation complications occasionally occur. Local anesthetic complications arise immediately or some minutes after the injection, and their consequences may be temporary or prolonged [1].

Among the complications after local anesthetic injections, the ocular complications probably represent a challenge for dentists, as the orbit is an anatomical structure with vessels and nerves distant from the dental practice [2]. For this reason, while some dentists may be experienced in the management of oral complications after anesthetic injection, like bleaching of the injected site or cardiovascular complications like tachycardia or syncope, the management of ocular complications after anesthetic injection may be difficult.

Ocular complications after dental anesthetic injection represent a rare occurrence in routine practice but impact dental practice significantly. Further, this complication makes it more difficult or impossible for the patient to be treated under local anesthesia, generating a situation of stress and anxiety for both the dentist and the patient. The management of a patient with ocular derangement may raise some difficulties.

A recent systematic review of a great number of papers reporting dental injections and ocular complications evidenced diplopia as the most common ocular complication after dental anesthesia, followed by ptosis and ophthalmoplegia [3].

Diplopia, also known as double vision, is a condition in which a person sees two images of a single object simultaneously. It occurs when the eyes are unable to align properly, leading to the perception of two distinct images. There are various causes of diplopia, including muscle weakness or paralysis in the eye muscles, nerve damage or compression, certain medical conditions such as diabetes or multiple sclerosis, trauma or injury to the eye or its surrounding structures, and certain medications [3]. The severity of diplopia can range from mild, intermittent episodes to constant and severe double vision. Double vision can significantly impact a person’s daily activities, as it impairs their ability to focus, judge distances accurately, and perform tasks that require visual coordination. However, the underlying cause of diplopia can often be treated, leading to the improvement or resolution of double vision. Treatment options may include wearing special glasses or using eye patches to block one of the images, performing eye exercises to strengthen weakened muscles, medication to manage underlying conditions, or surgery in more severe cases. In cases of diplopia, it is important to consult with an optometrist or ophthalmologist to determine the cause and appropriate treatment options [4].

The two most common injection techniques that are involved in ophthalmologic complications are the inferior alveolar nerve block (IANB) and the posterior superior alveolar nerve block [5,6]. The inferior alveolar nerve block (IANB) technique is a common method used to achieve anesthesia of the lower teeth, lower lip, and chin. The step-by-step description of the IANB technique provides correct positioning, as the patient is usually positioned upright or slightly reclined in the dental chair. The preparation and anesthetic solution provide that the operator prepares the anesthetic solution, typically lidocaine or articaine with a vasoconstrictor like epinephrine, in a sterile syringe and ensures proper patient preparation, including explaining the procedure, obtaining informed consent, and addressing any concerns. With regards to the technical note, the patient is asked to keep their mouth open wide enough for access to the injection site, and the operator locates the pterygomandibular raphe, a fold of tissue on the inside of the patient’s cheek near the uppermost part of the mandible, where the needle will be inserted. The injection site is cleaned and disinfected using an antiseptic. A topical anesthetic gel or spray may be applied to the injection site to reduce discomfort during needle insertion. Using a sterile, disposable dental syringe with an appropriately sized needle (usually 25–27 gauge), the operator enters the patient’s mouth with the needle, placing it near the pterygomandibular raphe, and the needle is directed slightly upwards, towards the coronoid notch, at a 45-degree angle to the occlusal plane. At this point, the operator advances the needle slowly until it reaches the lingual bone, which indicates it is near the inferior alveolar nerve, located within the mandibular canal. Before injecting the anesthetic solution, the operator aspirates to ensure the needle is not in a blood vessel. If no blood is aspirated, the anesthetic solution is slowly injected near the nerve in milliliter increments. After the injection, the needle is carefully withdrawn from the injection site. Afterwards, the dentist monitors the patient for any signs of complications or adverse reactions. The patient is given instructions for post-operative care, including what to expect and how to manage any post-operative discomfort. It is important to note that the IANB technique requires a thorough understanding of the anatomy and clinical experience to minimize the risk of complications [5].

The most common ocular complication after anesthetic injection reported by the patients is diplopia. However, facial nerve palsy, too, has been reported as a complication after IANB [5]. Facial nerve palsy is a condition that affects the facial nerve, which controls the movement of the muscles responsible for facial expressions. It often occurs suddenly, typically on one side of the face, and can result in weakness or paralysis of the affected muscles. Symptoms of unilateral facial nerve palsy are due to the muscles involved by the dental anesthetic injection, as orbicularis oculi, levator labii, and zygomaticus are the muscles that, with a facial palsy, have a great visual impact.

The most common symptoms of immediate facial nerve palsy include: (1) weakness or paralysis, as the ability to control the facial muscles on one side of the face is impaired, leading to difficulty in making facial expressions, such as smiling or closing the eye on the affected side; (2) drooping of the face, as the muscles on the affected side may droop, causing one side of the face to appear lower than the other; (3) loss of taste, as some people with facial nerve palsy may experience a distorted sense of taste or a loss of taste on the front two-thirds of the tongue; (4) eyelid and tear problems, as the affected eye may have trouble closing completely, leading to dryness and irritation; (5) speech difficulties, as in some cases, facial nerve palsy can affect the muscles involved in speech production, resulting in slurred or difficult-to-understand speech.

Bell’s palsy is a peripheral facial nerve palsy due to an undetectable cause, while secondary peripheral facial nerve palsy after dental anesthetic injections recognizes a direct etiology and is commonly due to the spread of the anesthetic solution towards facial nerve fibers [6]. The explanation of the spread of the anesthetic solution and its pathway to the trigger point to evoke the facial nerve palsy is challenging for clinicians. We report a case of facial nerve palsy after dental anesthesia with an unusual presentation, and the reasonable pathways of the anesthetic solution are discussed.

## 2. Case Report

**Figure 1:**
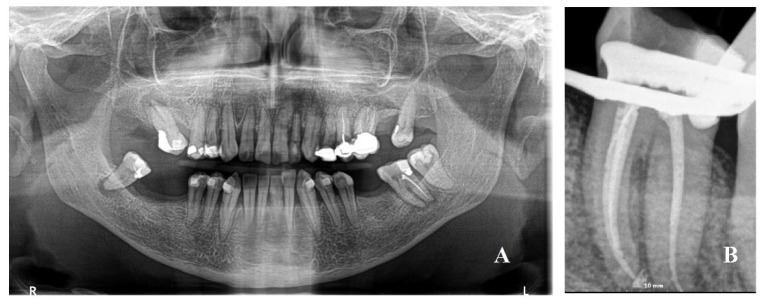
(**A**) Panoramic X-ray with a periapical lesion of the left mandibular second molar with mandibular foramen very posterior; (**B**) periapical X-ray after the endodontic retreatment.

A Caucasian 60-year-old woman (American Society of Anesthesiologists (ASA) I classification) under orthodontic treatment with aligners was referred by her dentist for severe pain on her left second molar in May 2022. She had no history of systemic disease. The patient presented an acute onset of pain that increased with chewing. On the panoramic X-ray, an apical radiolucency to the second molar tooth was identified, along with an incomplete previous root canal treatment. A periapical lesion was identified as responsible for the severe pain of the emergency visit. The emergency treatment provided ibuprofen 600 mg 1× every 12 h until pain decreased and amoxicilline 875 + 125 mg every 12 h each day for 6 days to avoid chewing with the left side; lower aligners were modified on the left second molar. Endodontic retreatment was scheduled 10 days after the visit (Figure 1).

**Figure 2:**
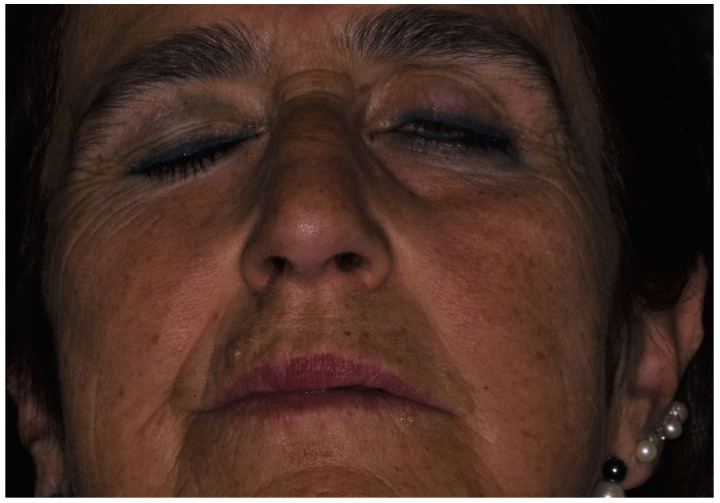
Image of the patient at the onset of facial nerve palsy and lagophtalmus with a Bell’s phenomenon.

At the endodontic date, an inferior alveolar nerve block (IANB) on the left side was performed with 1.8 mL of optocaine with epinephrine 1:200,000 and a disposable needle measuring 25 G × 36 mm mounted on a dental syringe was used. The effect of the anesthesia arose after 10 min. A rubber dam was mounted, and the procedure was completed in 1 and a half hours. At the end of the date, the patient reported feeling a facial palsy, with a physical examination that showed the asymmetry of the left naso-labial fold and left lip (Figure 2). 

**Figure 3:**
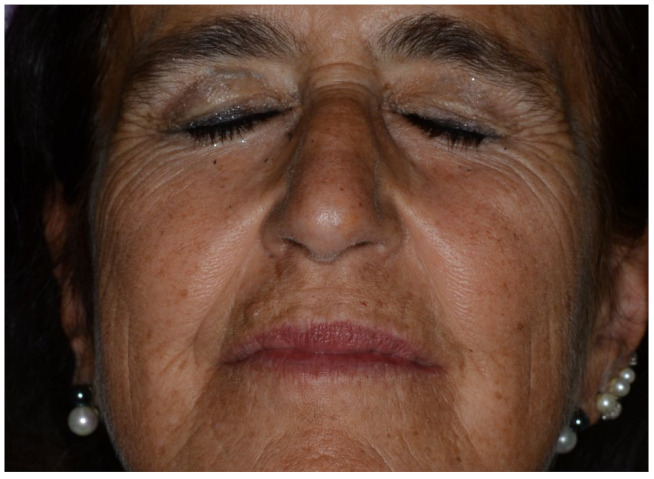
Recovery of the patient with correct eye closure.

The patient was immediately visited by an ophthalmologist. A lagophtalmus with a normal Bell’s phenomenon was also revealed. There was no evidence for epiphora or conjunctivitis. No diplopia or ocular motility dysfunctions were present. No other neurological deficit was found. A diagnosis of mild-moderate dysfunction of the seventh cranial nerve or grade IV of the House–Brackmann grading system for facial paralysis, possibly due to the local anesthetic injection, was made. Immediate pharmacological treatment was performed with Metilprednisolon 16 mg for 6 days with a decreasing dosage; the patient was invited to rest in the waiting room until the end of the anesthesia that occurred after 4 h. The patient recovered 4 h after the injection and was visited the following day and 30 days afterwards. The following day, no ocular dysfunction of facial palsy was present, nor were there any 30 days after the complication (Figure 3).

## 3. Discussions 

Iatrogenic peripheral facial nerve palsy represents a challenging complication for the general dentist, with different clinical symptoms that may lead the clinician into pitfalls.

An impairment of the facial nerve results in facial asymmetry, dry eyes, a reduction in saliva flow, and a loss of taste sensitivity in the anterior two-thirds of the tongue and the palate. The severity of the facial impairment may be assessed according to the extent of the motor deficiency with clinical scales, also allowing it to be monitored. The House-Brackmann scale is most often used.

On the basis of the time elapsed from the anesthetic injection to the onset of the clinical symptoms, the facial nerve palsy could be immediate or delayed, with different pathological pathways and times of clinical appearance.

The immediate type of peripheral facial nerve palsy starts just after the anesthetic injection and recovers a few hours later. This type of palsy happens more likely when direct contact with the anesthetic solution of one or more fibers of the facial nerve is realized. This palsy may happen when the anesthetic solution is injected too far posteriorly and the solution is delivered in the parotid substance, where the parotid lobe is present near the posterior and medial surfaces of the mandibular ramus. The parotid gland often incorporates facial nerves, and this could lead to transient facial palsy. However, some authors underline the difficulty of performing anesthesia of the facial nerve through the oral cavity, as this method is poorly performed [7]. On the other hand, the development of other anesthetic techniques in wisdom teeth surgery reduces the use of IANB in order to reduce anesthetic complications [8,9]. In the present case, the IANB was successful, so the technique resulted was correctly performed. Anyway, a possible anatomical variation of the facial nerve outside the parotid gland previously reported in the literature or the presence of aberrant facial nerve fibers near the retromandibular space could make this complication more likely, even if the IANB technique is correctly performed [10,11,12].

The delayed type of peripheral facial nerve palsy could be evoked by many different etiologies instead. A first hypothesis takes into account the possibility that the injection itself or the local metabolism of the anesthetic drug generated a sympathetic vascular reflex with a consequent ischemic paralysis of the region of the mastoid foramen. This stimulus of the sympathetic plexus is associated with the external carotid artery, which communicates with the plexus through the stylomastoid artery, which enters the parotid gland and hence arrives at contact with the facial nerve. This sympathetic plexus stimulation could cause delayed stimulation of the vasa nervorum of the facial nerve with secondary ischemic neuritis and edema [13,14].

Another hypothesis, less probable given the lack of a remote pathological history of infection, could be the reactivation of herpes simplex virus (HSV) or varicella-zoster virus (VZV) following the trauma associated with the injection of anesthesia. This event could cause specific neural inflammation and the following facial nerve palsy [13]. Schirm and Mulkens [7] suggested that the re-activation of HSV genomes in the geniculate ganglia is the most important etiology of Bell’s palsy. With regards to VZV re-activation and peripheral facial nerve palsy, this syndrome has been known as Ramsay Hunt syndrome and zoster sine herpete; in fact, zoster sine herpete lacks present vertigo, herpetic epithelial lesions, or hearing loss and is often clinically diagnosed as Bell’s palsy. A serologic assay is mandatory to detect VZV reactivation [15].

Another hypothesis of delayed facial nerve palsy after IANB could be the intravascular backward flow of the anesthetic solution via an intra-arterial injection. This method has been reported many times, both for superior and inferior alveolar nerve injections [3,5,16].

Another possible etiology of delayed facial nerve palsy in the present case could be due to the prolonged time for endodontic re-treatment (one hour and a half). This prolonged time, even if not exaggerated in molar endodontics, could be the cause of a possible stretch of the facial nerve due to prolonged instrumental opening of the mouth with a rubber dam [17,18]. This aspect, the prolonged instrumental opening of the mouth, is commonly an etiology for temporomandibular joint disorders, too [19]. 

In delayed palsy, the onset of symptoms is delayed for a few hours to a few days, and the course is more protracted [5]. Keetley et al. [20] reported 0.3% of facial nerve palsy after IANB in their paper. However, they suggest that facial palsy could be more common due to reduced symptoms. Clinical aspects that may reduce the evaluation of facial palsy symptoms may be the use of injections after general anesthesia with a stunned patient that has fewer symptoms, like the case of Jenyon et al. [21]. Another clinical condition may be subsequent palsy but not late onset, like the one reported in the present paper, a variant modality of palsy with aspects of immediate and late onset. The immediate palsy with a short duration during a visit with the use of the rubber dam or during conscious sedation may be another clinical aspect that may reduce palsy evaluation, too.

The position of the patient may have a role, too. The injection with a long needle in the very posterior position of the mandibular foramen could spread the anesthetic solution behind the mandibular ramus but inside the parotid loggia; often the parotid gland envelopes the facial nerve, thus leading to the direct anesthesia of the latter.

In the present case, the interesting point, the subsequent onset, could be related to a late effect of the anesthetic solution due to the interference of the parotid gland, as anomalies of salivary production could not be evidenced in a few hours. Further, the IANB effect arose after 10 min, as usual, suggesting that some of the solution went to the mandibular foramen and some went to parotid loggia. Needle aspiration in this case was performed, but without a significant answer. Some authors [7] cite the difficulty of anesthetizing the facial nerve through the oral cavity, making this mechanism unlikely. However, there are deviations from normal anatomy that increase the chances of direct exposure to local anesthetic solutions, even if the anesthesia is administered properly [3,13].

Facial nerve palsy represents a puzzling complication for the general dentist. For this reason, some practitioners use ligamental injections to reduce these complications in IANB [8,9]. In cases of ocular complications after dental anesthesia, a multidisciplinary group should study the patient and the condition to evaluate the class of these complications and the prognosis. Patients should be informed of the possibility of an anatomical deviation, and alternative anesthetic techniques should be considered in these patients. 

## Data Availability

The data presented in this study are available in this article.
